# The Effects of Hemodynamic Changes on Pulse Wave Velocity in Cardiothoracic Surgical Patients

**DOI:** 10.1155/2016/9640457

**Published:** 2016-11-09

**Authors:** Yurie Obata, Maki Mizogami, Sarabdeep Singh, Daniel Nyhan, Dan E. Berkowitz, Jochen Steppan, Viachaslau Barodka

**Affiliations:** ^1^Division of Cardiac Anesthesia, Department of Anesthesiology and Critical Care Medicine, The Johns Hopkins University School of Medicine, Baltimore, MD, USA; ^2^Department of Anesthesiology and Reanimatology, University of Fukui, Fukui, Japan

## Abstract

The effect of blood pressure on pulse wave velocity (PWV) is well established. However, PWV variability with acute hemodynamic changes has not been examined in the clinical setting. The aim of the present study is to investigate the effect of hemodynamic changes on PWV in patients who undergo cardiothoracic surgery. Using data from 25 patients, we determined blood pressure (BP), heart rate (HR), and the left ventricular outflow tract (LVOT) velocity-time integral. By superimposing the radial arterial waveform on the continuous wave Doppler waveform of the LVOT, obtained by transesophageal echo, we were able to determine pulse transit time and to calculate PWV, stroke volume (SV), cardiac output (CO), and systemic vascular resistance (SVR). Increases in BP, HR, and SVR were associated with higher values for PWV. In contrast increases in SV were associated with decreases in PWV. Changes in CO were not significantly associated with PWV.

## 1. Introduction

Pulse wave velocity (PWV) is one of the most widely used surrogates of arterial stiffness [1]. Previous studies demonstrated that PWV is associated with various factors such as age, gender, salt intake, genetic factors, blood pressure (BP), and heart rate (HR) [2, 3]. Both elevated PWV and elevated BP have been associated with negative cardiovascular outcomes [4]. However, in most of these studies, PWV were obtained at rest and with stable hemodynamics. The extent of immediate PWV variability with acute hemodynamic changes in humans remains unknown. Animal studies that used invasive techniques suggested that PWV, similar to BP, undergoes rapid changes with acute changes in hemodynamics [5]. The purpose of the present study is to examine the effect of acute hemodynamic changes on PWV by measuring pulse transit time from the continuous wave (CW) Doppler waveform and the radial arterial waveform in patients undergoing cardiothoracic surgery. This approach allows the simultaneous measurement of pulse transit time, BP, heart rate, stroke volume, cardiac output, and peripheral vascular resistance for the same heartbeat.

## 2. Materials and Methods

### 2.1. Subjects

This retrospective observational study was based on the images from our cardiac surgery intraoperative transesophageal echocardiography (TEE) database at The Johns Hopkins Hospital, Baltimore, Maryland, between October 2011 and September 2013. The protocol was approved by The Johns Hopkins Medicine Institutional Review Boards (IRB00088711).

The study included patients, 18 years of age or older, undergoing cardiothoracic surgery who had at least three images stored in the database on which the radial arterial waveform was superimposed on the CW Doppler waveform of left ventricular outflow tract (LVOT). All images were obtained before the initiation of cardiopulmonary bypass (CPB) and with different values for BP. Patients with arrhythmias or implanted cardiac pacemaker were excluded.

### 2.2. Measurement

**Figure 1:**
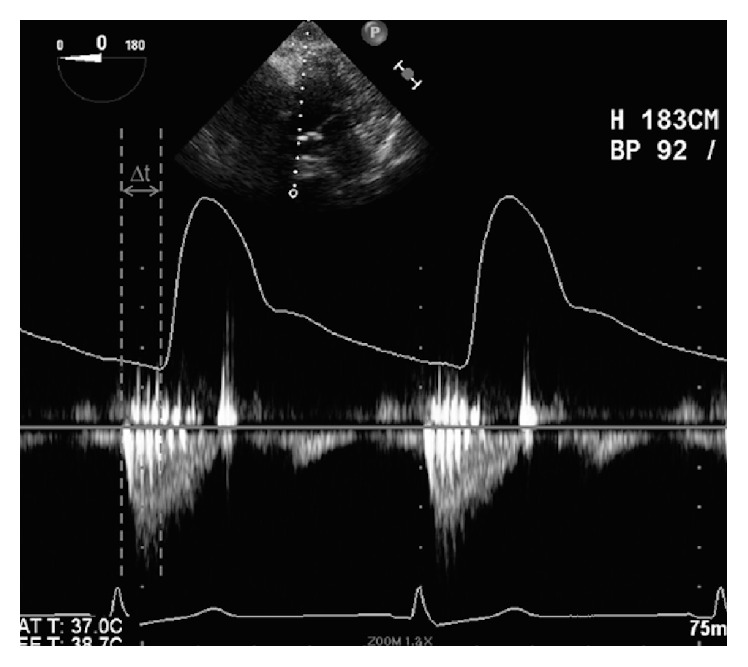
Representative image of measurements. We measured the transit time (Δ*t*) from the start of ejection on continuous wave Doppler waveform to the upstroke on the radial arterial waveform.

Intraoperative care was similar in all patients, all of which received general anesthesia with a combination of midazolam (2–10 mg), fentanyl (750–2000 *μ*g), vecuronium (10–20 mg), and isoflurane (0.5–1%) after 20-gauge radial arterial catheter insertion for continuous BP measurements. We use right radial artery as a primary site for arterial cannulation. A transesophageal echo (TEE) probe was inserted after the induction of general anesthesia and a comprehensive TEE examination performed by a certified cardiac anesthesiologist using a Philips iE33 ultrasound machine (Philips Medical Systems, Amsterdam, Netherlands). The arterial pressure and electrocardiogram (ECG) waveforms were recorded and simultaneously projected onto the LVOT CW Doppler waveform by connecting the clinical monitor (GE Healthcare, Little Chalfont, UK) to the TEE machine, at the identical speed of 25 mm/s (Figure 1). The CW Doppler images were obtained from either the deep transgastric or transgastric long axis views of the aortic valve. All images were stored on the clinical server and the offline measurements performed using the software “Synapse Cardiovascular” (FUJIFILM, Tokyo, Japan). We defined pulse transit time (Δ*t*) as the time from the foot of the CW Doppler waveform (start of ejection) to the origin of the upstroke on the arterial waveform (Figure 1). PWV was obtained by dividing vascular path length (*L*) by Δ*t*. The vascular path length is the distance traveled by the pulse wave from the aortic valve to the site of the radial artery catheter. We estimated the vascular path length using the formula *L* = (demi-span) − (hand  length), where demispan (distance from sternal notch to the tip of the fingers) and hand length were estimated from height, age, and gender as described previously [6, 7]. Stroke volume (SV) was calculated using the formula SV = LVOT  CSA × LVOT  VTI, where CSA is the cross-sectional area of the LVOT and VTI is velocity-time integral across the LVOT. LVOT VTI was measured by tracing the CW Doppler waveform. In cases were more than one beat per image was captured, Δ*t* and VTI were averaged across both beats. Calculation of the LVOT CSA was performed by measuring the LVOT diameter from the midesophageal long axis view. The systolic, diastolic, and pulse pressure were measured from the simultaneously recorded arterial blood pressure waveform. The mean arterial pressure (MAP) was calculated according to the following formula: MAP = DBP + PP/3, where DBP is diastolic blood pressure and PP is pulse pressure. HR was determined from the ECG signal, recorded on the same TEE image. Cardiac output (CO) and systemic vascular resistance (SVR) were calculated as follows: CO = SV × HR and SVR = 80 × MAP/CO.

### 2.3. Statistical Analysis

We report continuous variables as mean ± standard deviation (SD) or as median (interquartile range, IQR) and categorical variables as proportions. The relationship between two continuous variables was assessed using a simple linear regression analysis (SBP (systolic blood pressure), MAP, DBP, PP, HR, SV, CO, and SVR) and PWV within each patient. To account for within-subject (or patient) variability on estimation of the effect of the BP, HR, SV, CO, and SVR on PWV, a linear mixed model was created [8]. Due to high multicollinearity (Variance Inflation Factor (VIF) > 10) among the blood pressure variables (MAP, SBP, PP, and DBP), we decided to consider only MAP as a predictor variable. Similarly, we found high multicollinearity Variance Inflation Factor ((VIF) > 10) among SV, CO, and SVR variables. Due to multicollinearity between SV, CO, and SVR, we decided to consider MAP, HR, and SV variables in Model A; MAP, HR, and SVR in model B; MAP and CO in model C to study the association between outcome variable (PWV) and predictor variables after adjusting for age and body mass index (BMI) of the patients. In order to choose the best model, Akaike Information Criterion (AIC) with the smaller-is-better criterion was used [9]. The linear mixed model analysis was performed in R version 3.2.2 (R foundation for Statistical Computing, Vienna, Austria) [10]. The rest of the analysis was performed with GraphPad Prism version 6.0 (GraphPad Software, La Jolla, California, USA). Statistical significance was set at *P* < 0.05 and all tests were two-sided.

## 3. Results

A total of 237 samples from 25 patients were included in the analysis. The median number of beats per patient was 8 (interquartile range (IQR): 5 to 15). No arrhythmias were presented in the beats analyzed. The baseline characteristics of the patients are outlined in Table 1. None of the patients required a continuous infusion of an inotropic drug or mechanical cardiopulmonary support before the planned initiation of cardiopulmonary bypass (CPB). The median vascular path length was 63.76 cm (IQR: 60.21 cm to 64.42 cm).

**Figure 2:**
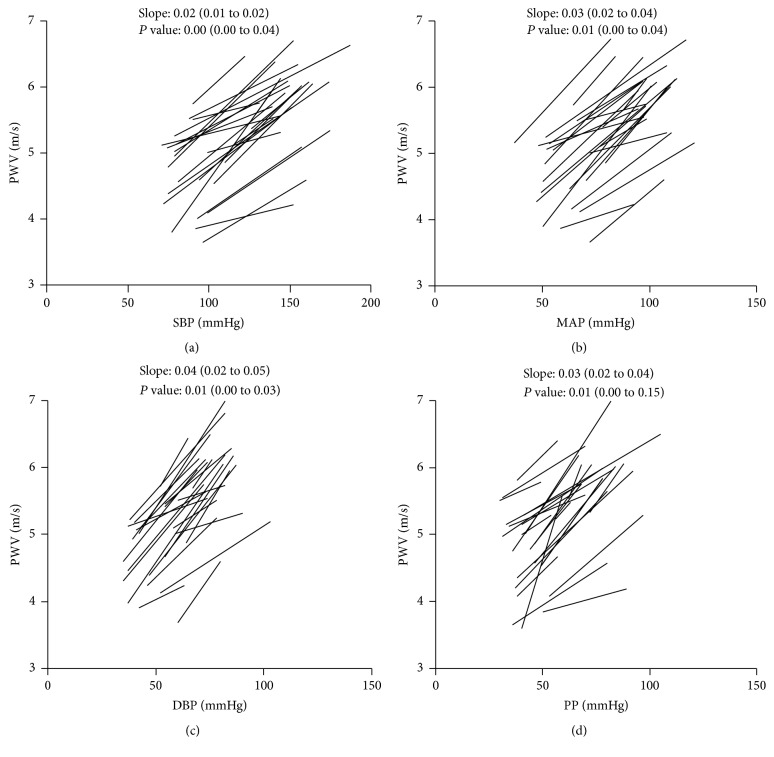
The relationship between PWV and BP. Each line indicates a simple linear regression line within subject. (a) The relationship between PWV and SBP. (b) The relationship between PWV and MAP. (c) The relationship between PWV and DBP. (d) The relationship between PWV and PP. The median (IQR) slope and the median (IQR)* P* value are presented in each graph. BP: blood pressure, DBP: diastolic blood pressure, IQR: interquartile range, MAP: mean arterial pressure, PP: pulse pressure, PWV: pulse wave velocity, and SBP: systolic blood pressure.

There was a significant correlation between PWV and BP (SBP, MAP, DBP, and PP) within individual patients (Figure 2). Given that each patient had a different linear regression line with a different* P* value, the overall trend for all regression lines was estimated by a linear mixed model and presented in Table 2. A positive correlation was observed between PWV and SBP (slope: 0.016, *P* < 0.001), DBP (slope: 0.03, *P* < 0.001), PP (slope: 0.026, *P* < 0.001), and MAP (slope: 0.024, *P* < 0.001).

**Figure 3:**
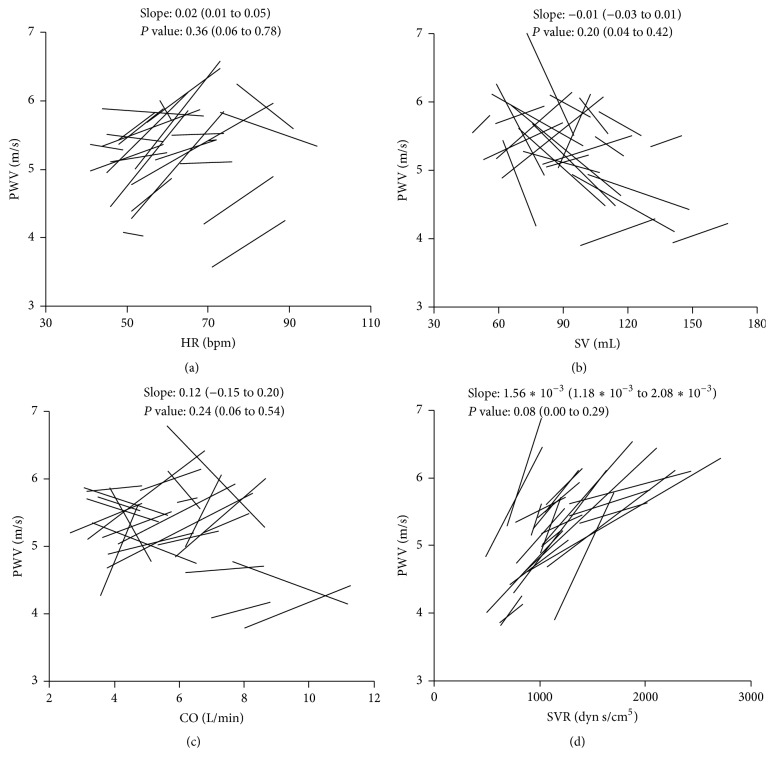
The relationship between PWV and HR, SV, CO, and SVR. Each line indicates a simple linear regression line within subject. (a) The relationship between PWV and HR. (b) The relationship between PWV and SV. (c) The relationship between PWV and CO. (d) The relationship between PWV and SVR. The median (IQR) slope and the median (IQR)* P* value are presented in each graph. CO: cardiac output, HR: heart rate, IQR: interquartile range, PWV: pulse wave velocity, SV: stroke volume, and SVR: systemic vascular resistance.

The linear regression lines demonstrate a relationship between PWV and HR, PWV and SV, PWV and CO, and PWV and SVR within individual patients (Figure 3). Given that each patient had a different linear regression line with a different* P* value, the overall trend for all regression lines was estimated by a linear mixed model and presented in Table 2. The estimated slope showing a relationship between PWV and HR was 0.026 (*P* < 0.001), between PWV and SV was −0.007 (*P* < 0.01), between PWV and CO was 0.05 (*P* > 0.05), and between PWV and SVR was 0.001 (*P* < 0.001) (Table 2). Hence, the increase in PWV was significantly associated with an increase in both HR and SVR. The effect of SV was opposite to the one of HR and SVR. An increasing SV corresponded to decreased PWV. Changes in CO were not significantly associated with changes in PWV.

Linear mixed model A showed that PWV is positively correlated with MAP and negatively correlated with SV; however, PWV was not correlated with HR (Table 3). Liner mixed model B showed that PWV is positively correlated with MAP, HR, and SVR and negatively with BMI. Linear mixed model C showed that PWV is positively correlated only with MAP; however, PWV was not correlated with CO. Between the 3 models considered, the lowest AIC of 131.22 was observed for model A.

## 4. Discussion

In the present study, we confirm that acute hemodynamic changes result in changes in PWV. Our results support published data obtained in animal studies showing that PWV is strongly affected by MAP, such that increasing MAP lead to a corresponding increase in PWV [11]. From a clinical perspective, MAP changes with HR, SV, or SVR. To the best of our knowledge, there are no prior clinical studies that determine which specific components associated with blood pressure (HR, SV, or SVR) are responsible for the observed changes in PWV. Our measuring method enabled us not only to simultaneously determine pulse transit time and BP but also to calculate SV, CO, and SVR for the same heartbeat. Our results clarified the relationships among BP, HR, SV, CO, SVR, and PWV in the patients who underwent cardiothoracic surgery and showed that in addition to changes in MAP changes in SVR and HR are responsible for the observed changes in PWV. Interestingly, changes in CO by itself did not explain changes in PWV. Moreover, increases in SV were associated with decreases in PWV. These findings strongly suggest that increases in SVR and HR are mainly responsible for the observed increases in PWV with increased MAP.

The main challenge to accurately assess the effects of the different hemodynamic parameters on PWV is to measure all of them simultaneously and continuously for the same heartbeat. For noninvasive measurements, the most popular way of real time PWV acquisition is based on the pulse transit time measured from the R-peak of the QRS complex of the electrocardiogram (ECG) to the upstroke of the peripherally derived arterial waveform [12]. Consequently, the measured time includes not only the pulse transmission interval, but also the delay due to intracardiac events such as electrical depolarization, isovolumic contraction, the opening of the aortic valve, and the expulsion of the blood [13]. These noninvasive techniques may introduce significant errors to the real pulse transit time measurements. To overcome these measurement errors, we defined pulse transit time as the time from the start of the ejection on the CW Doppler waveform to the upstroke on the radial arterial waveform.

We reported previously that PWV increases with rising MAP in young and old rats [5]. Similar results were observed in healthy male subjects by Stewart et al. [14]. They investigated the relationship between mean change in PWV and mean changes in MAP by administrating cardiovascular agents (dobutamine, norepinephrine). There was a close association between MAP and PWV such that increases in MAP corresponded to increases in PWV. Our finding of PWV dependence on MAP is consistent with that of previous studies.

Given that BP is related to CO and SVR by the equation BP = CO × SVR and CO is related to SV and HR, we investigated the relationships between PWV and HR, PWV and SV, PWV and CO, and PWV and SVR to assess the individual contributions of HR, SV, and SVR, in order to explain the relationship between MAP and PWV.

The effect of HR on PWV over a range of MAP has been examined in rats by Tan et al. [15]. Similar to our findings, their results demonstrated that PWV increases with increasing HR. However, the effect of HR on PWV was MAP dependent. In our present study, PWV increased with increase in HR, but the relation was not significant after accounting for multiple variables in a mixed model A, which was found to provide best fit based on AIC.

Kamoi et al. investigated the relationship between SV and PWV for various hemodynamic conditions in pigs [16]. They showed that changes in PWV corresponded to the preload dependent changes in SV. They thought it is due to the difference in the ratio of preload and afterload, influencing the value of PWV in individual subjects. Indeed, the relationship between PWV and SV was different in each individual patient in our study. However, our finding that increases in PWV are associated with decreases in SV possibly reflects the effect of SVR on MAP and through MAP on PWV. It has been described that pharmacologic increases in SVR lead to decrease in HR, SV, and CO despite increases in MAP [17].

In contrast to the varying effects of HR, SV, and CO on PWV in individual patients, the effect of SVR on PWV was consistent and similar to the effect of MAP such that PWV increased with increasing SVR in all patients. A study by Greene and Gerson supports these findings, as they are describing that acute and reversible changes in SVR are linearly positively related to arterial PWV in the anesthetized dog [18]. They implied that the mechanisms of changes of PWV with SVR are due to changes in arterial elasticity, dimensions as well as increases in local vascular impedance, and pressure wave reflection coefficients. However, PWV depends not only on intrinsic arterial wall stiffness, but also on wall tension. Wall tension in turn, depends on wall thickness, radius, vascular smooth muscle tone, and distending BP [19]. Our findings suggested that one reason for increasing PWV with increases in BP can be attributed to SVR rather than CO or its components (SV and HR).

The present study has several limitations. Firstly, the subjects studied represent a quite homogenous group of overweight males who underwent cardiothoracic surgery. These patient characteristics are known to affect vascular function and stiffness [20, 21]. Second, the data was collected on anesthetized and intubated patients during cardiothoracic surgery. Surgical stimulation, mechanical ventilation, volume status changes, and the effects of medications all affect vascular tone, SVR, SV, ventricular contractility, BP, and HR. As such the varying degree of the observed association among the variables might be confounded by those factors. Third, there are inaccuracies in estimating the vascular pass length, since we did not measure it directly in each patient but rather calculated it from height, age, and gender. However, the PWV variability within each subject depends on pulse transit time. That is why we assessed the relationship between PWV and hemodynamics accounted for individual subject variability. Finally, we measured aortic-radial PWV, not carotid-femoral PWV, which is the current gold standard of arterial stiffness measurement. Recently some studies reported the differences between PWV measured in central elastic versus muscular peripheral arteries [22]. However, similar to previous reports in which PWV was measured in central artery, we found that aortic-radial PWV varied with BP, HR, SV, and SVR.

## 5. Conclusions

The simultaneous recording of CW Doppler waveform and radial arterial waveform allowed us to shed light on how certain hemodynamics parameters affect PWV. PWV increases with rising BP, SVR, and HR and decreases with rising SV. CO has no effect on PWV in patients who underwent cardiothoracic surgery.

## Figures and Tables

**Table 1:** Demographic data and characteristics of 25 patients treated for the study.

Variable	Data
Number of patients	25
Age (years old)	
Median	67
IQR	59–71
Gender	
Female (%)	1 (4)
Male (%)	24 (96)
Height (cm)	
Median	175
IQR	168–176
BMI (kg/m^2^)	
Median	29
IQR	25–34
Operation	
Isolated CABG (%)	17 (68)
Valve (%)	3 (12)
CABG plus valve (%)	1 (4)
Others (%)	4 (16)

BMI: body mass index, CABG: coronary artery bypass grafting, and IQR: interquartile range.

**Table 2:** Estimates of intercept and slopes for dependent-variable~independent-variable after using linear mixed model.

Linear mixed models				
Variables	Intercept	95% CI (intercept)	Slope	95% CI (slope)
PWV~SBP	3.35^*∗∗∗*^	3.04, 3.65	0.016^*∗∗∗*^	0.01, 0.02
PWV~MAP	3.29^*∗∗∗*^	2.98, 3.60	0.024^*∗∗∗*^	0.02, 0.03
PWV~DBP	3.25^*∗∗∗*^	2.95, 3.55	0.03^*∗∗∗*^	0.029, 0.036
PWV~PP	3.76^*∗∗∗*^	3.44, 4.08	0.026^*∗∗∗*^	0.02, 0.03
PWV~HR	3.65^*∗∗∗*^	3.06, 4.26	0.026^*∗∗∗*^	0.01, 0.03
PWV~SV	6.00^*∗∗∗*^	5.51, 6.49	−0.007^*∗∗*^	−0.01, −0.002
PWV~CO	4.97^*∗∗∗*^	4.49, 5.46	0.05	−0.02, 0.12
PWV~SVR	3.86^*∗∗∗*^	3.55, 4.16	0.001^*∗∗∗*^	0.001, 0.0014

^*∗∗∗*^ *P* value < 0.001; ^*∗∗*^ *P* value < 0.01.

CI: confidence interval; CO: cardiac output; HR: heart rate; PP: pulse pressure; PWV: pulse wave velocity; SBP: systolic blood pressure; SV: stroke volume; SVR: systemic vascular resistance; MAP: mean arterial pressure; DBP: diastolic blood pressure.

**Table:** (a) Model A: estimated coefficients, 95% CI, and *P* value after using linear mixed model on PWV~Age+BMI+MAP+HR+SV

Variables	Estimated coefficients	95% CI	*P* value
Age	0.007	−0.006, 0.021	0.30
BMI	−0.03	−0.06, −0.0006	0.06
MAP	0.026	0.022, 0.028	<0.001
HR	0.002	−0.003, 0.008	0.47
SV	−0.01	−0.13, −0.007	<0.001

BMI: body mass index; CI: confidence interval; HR: heart rate; MAP: mean arterial pressure; SV: stroke volume.

**Table:** (b) Model B: estimated coefficients, 95% CI, and *P* value after using linear mixed model on PWV~Age+BMI+MAP+HR+SVR

Variables	Estimated coefficients	95% CI	*P* value
Age	0.007	−0.007, 0.022	0.35
BMI	−0.035	−0.07, −0.003	0.04
MAP	0.014	0.01, 0.018	<0.001
HR	0.017	0.01, 0.02	<0.001
SVR	0.0007	0.0004, 0.0009	<0.001

BMI: body mass index; CI: confidence interval; HR: heart rate; MAP: mean arterial pressure; SVR: systemic vascular resistance.

**Table:** (c) Model C: estimated coefficients, 95% CI, and *P* value after using linear mixed model on PWV~Age+BMI+MAP+CO

Variables	Estimated coefficients	95% CI	*P* value
Age	0.008	−0.007, 0.023	0.32
BMI	−0.023	−0.056, 0.009	0.19
MAP	0.023	0.01, 0.029	<0.001
CO	−0.046	−0.13, 0.04	0.31

BMI: body mass index; CI: confidence interval; CO: cardiac output; MAP: mean arterial pressure; SVR: systemic vascular resistance.
